# Neurophysiological evidence of motor contribution to vicarious affective touch

**DOI:** 10.1093/cercor/bhae441

**Published:** 2024-11-06

**Authors:** Niccolò Butti, Cosimo Urgesi, Stergios Makris, Francis P McGlone, Rosario Montirosso, Valentina Cazzato

**Affiliations:** Scientific Institute, IRCCS E. Medea, 0-3 Center for the at-Risk Infant, Via Don Luigi Monza 20, 23842 Bosisio Parini (LC), Italy; PhD Program in Neural and Cognitive Sciences, Department of Life Sciences, University of Trieste, Via Licio Giorgieri 5, 34127 Trieste, Italy; Scientific Institute, IRCCS E. Medea, Pasian di Prato, Via Cialdini 29, 33037 Pasian di Prato (UD), Italy; Laboratory of Cognitive Neuroscience, Department of Languages and Literatures, Communication, Education and Society, University of Udine, Via Margreth 3, 33100 Udine, Italy; Department of Psychology and Research Centre for Arts and Wellbeing, Edge Hill University, St Helens Road, Ormskirk, Lancashire L39 4QP, United Kingdom; Faculty of Science & Engineering, School of Life Sciences, Manchester Metropolitan University, All Saints Building, Manchester M15 6BH, United Kingdom; School of Science, Department of Neuroscience and Biomedical Engineering, Aalto University, Otakaari 24, 02150 Espoo, Finland; Scientific Institute, IRCCS E. Medea, 0-3 Center for the at-Risk Infant, Via Don Luigi Monza 20, 23842 Bosisio Parini (LC), Italy; School of Psychology, Faculty of Health, Liverpool John Moores University, Tom Reilly Building, Byrom Street, Liverpool L3 3AF, United Kingdom; Department of Cognitive, Psychological and Pedagogical Sciences, and Cultural Studies, University of Messina, Via Concezione 6, 98121 Messina, Italy

**Keywords:** vicarious affective touch, C-tactile afferents, motor resonance, single-pulse transcranial magnetic stimulation, motor-evoked potential

## Abstract

Understanding observed interpersonal touch, particularly the so-called affective touch targeting the CT fibers, is essential for social interactions. Research has documented that observing other people being touched activates the same cortical areas involved in direct tactile experiences. However, observing interpersonal touch also activates an inner simulation of the movements in the observer’s motor system. Given the social and affective significance of CT-optimal touch, the present study tested the hypothesis that observing stroking touches targeting or not targeting the CT fibers system might distinctly influence motor resonance to vicarious touch. With this aim, we used single-pulse transcranial magnetic stimulation and motor-evoked potentials recording while participants observed video clips of interpersonal touch events at different stroking velocities. We found a modulation of motor system activity, particularly a decrease in corticospinal excitability, when observing CT-optimal touch as opposed to non-CT-optimal velocities, a mechanism that might aid in understanding the touchee’s feelings during vicarious interpersonal touch. Moreover, participants with higher reliance on bodily cues to be emotionally aware showed greater motor suppression for CT-optimal compared to non-CT-optimal velocities. These results shed light on the complex interplay between motor and somatosensory systems in social touch perception and emphasize the importance of affective touch in human social interactions.

## Introduction

Understanding affective touch through the observation of actions such as handshaking, hugging, and caressing is essential for navigating social environments. Neurophysiological responses to tactile stimulation are mediated by distinct sensory systems: discriminative touch by fast, myelinated Aβ fibers, and gentle tactile stimulation by slow-unmyelinated C-tactile (CT) fibers (Vallbo et al. 1999; McGlone et al. 2014). The CT system, primarily located in the hairy skin of mammals, is optimally responsive to caress-like strokes at velocities of 1 to 10 cm/s and at temperatures akin to human skin (Olausson et al. 2010; Ackerley et al. 2014a). CT fiber activation follows an inverted U-shaped response curve in relation to stroke speed and evokes corresponding levels of pleasant sensations (Essick et al. 1999; Essick et al. 2010; Ackerley 2022). The term “affective touch” is thus used to describe stimulation that typically targets CT fiber activation. Human social stroking is naturally optimized for CT-optimal touch (Croy et al. 2016), serving as a soothing form of stimulation and supporting social bonding (Cascio et al. 2019). While direct recordings of CT fiber activation during vicarious touch are not available, it is hypothesized that observing such touch may be processed as a prioritized type of information (Morrison et al. 2011a; Pereira et al. 2023 Feb 28).

From a neuroanatomical perspective, CT afferent projections are directed to the posterior insular cortex (pIC), a brain region considered essential for emotion regulation and integration of inner bodily signals (Morrison 2016; Kirsch et al. 2020). Extensive research provides support for the functional role of CT fibers in social functioning and affects regulation across the lifespan (Cascio et al. 2019; Montirosso and McGlone 2020; Croy et al. 2022), with evidence of insular activation to caress-like touch being observed in the early stages of infant life (Jönsson et al. 2018; Tuulari et al. 2019). Beyond pIC, interpersonal touch activates somatosensory cortices (S1 and S2) and key areas of the social brain, such as the medial prefrontal cortex, the dorsal anterior cingulate cortex, and the inferior frontal gyrus (IFG), that contribute to the processing of affective dimensions of touch (Gordon et al. 2013; Case et al. 2017; Boehme et al. 2019).

Observing other people being touched appears to activate the same cortical areas involved in direct tactile experiences (Keysers et al. 2004; Keysers et al. 2010; Bolognini et al. 2012; Lee Masson et al. 2018). Within this network, IFG, the insula, and the superior temporal sulcus would aid in recognizing others’ emotions (Peled-Avron et al. 2019; Saporta et al. 2022; Schaefer et al. 2023), the temporoparietal junction would differentiate self from others (Lee Masson et al. 2020), and the somatosensory cortices would map the observed touch into an internal representation (Bolognini et al. 2013; Bellard et al. 2023). This visuo-tactile mirror mechanism, which is detectable in infants as young as 4 months old (Rigato et al. 2019a), would mimic the tactile stimulation and enable empathy for the other person’s sensations (Schaefer et al. 2012; Schaefer et al. 2013; Smit et al. 2023). Moreover, a functional relationship between activation in the pIC and processing of vicarious affective touch has been observed, suggesting that the brain is adept at distinguishing CT-targeted strokes when observing interpersonal touch events (Morrison et al. 2011a).

Within the “embodied simulation framework” (Gallese and Sinigaglia 2011), observing interpersonal touch actions can also activate an inner simulation of the movements in the observer’s motor system (Gazzola and Keysers 2009; Gallese and Ebisch 2013). For example, previous electroencephalography (EEG) research has reported a modulation of the mu and Rolandic rhythms during observation of interpersonal touch, indicating sensorimotor simulations that aid in understanding another’s tactile experience (Peled-Avron et al. 2016; Schirmer and McGlone 2019; Addabbo et al. 2020). This modulation is also seen in the toucher when administering a consoling touch, suggesting a connection between delivering touch and simulating another’s mental states (Peled-Avron et al. 2018). However, it remains to be determined whether observing affective interpersonal touch, particularly those targeting CT-optimal strokes, selectively influences motor cortex activation and supports the understanding and prediction of the consoling intention underlying observed stroking (Avenanti et al. 2018; Paracampo et al. 2018).

The phenomenon of motor resonance, a widely acknowledged index of motor simulation, involves mapping others' actions onto one's own motor repertoire. It reflects the activation of the motor system during action observation (for a comprehensive review, see Fadiga et al. 2005; Pineda 2008; Avenanti et al. 2013; Naish et al. 2014), and contributes to action understanding and imitation (Pobric et al. 2006; Tidoni et al. 2013; Urgesi et al. 2014; Jacquet and Avenanti 2015). Motor resonance is often indicated by changes in motor-evoked potentials (MEPs), measured using electromyography (EMG) following transcranial magnetic stimulation (TMS) over the primary motor cortex (M1). These changes in MEPs, or corticospinal excitability (CSE), are thought to reflect the dynamics of motor facilitation or suppression, corresponding to the increase or decrease in motor simulation processes for the observed action (Liuzza et al. 2015; Amoruso et al. 2016). Interestingly, the simulative representation of observed actions encompasses not just movement kinematics but also their affective meanings (Craighero and Mele 2018; Vicario et al. 2019; Urgesi et al. 2020). Given the social and affective significance of CT-optimal touch, it is hypothesized that observing stroking targeting or not targeting the CT fiber system might distinctly influence motor resonance to vicarious affective touch.

To investigate this, we used single-pulse (sp) TMS and MEP recording while participants observed video clips of interpersonal touch events at different stroking velocities. The rationale behind this approach is that spTMS to M1 elicits MEPs in the contralateral target muscle, and these MEP amplitudes are influenced by action observation, reflecting muscle selectivity and the kinematic profile of the observed movement (Fadiga et al. 1995; Romani et al. 2005; Urgesi et al. 2006; Alaerts et al. 2009). In this study, MEPs were recorded in two muscles of the participants’ right arm and compared across different stroking velocities (0, 5, 30 cm/s) and two body sites (hairy skin, i.e. hand-dorsum, vs. glabrous skin, i.e. palm). We reasoned that, if CSE was specifically mapping affective touch, then we should expect motor responses to slow touch to deviate from both static and fast conditions. Conversely, if CSE maps only kinematic aspects of the observed movements (e.g. motion), then we would see a linear modulation of CSE with increasing velocities, with motor responses to slow touch being intermediate between the static and fast conditions. The lack of previous investigations made any predictions regarding the direction of this effect exploratory. On one hand, former studies have reported a modulation of the mu rhythm during the observation and execution of interpersonal touch (Peled-Avron et al. 2018; Addabbo et al. 2020), suggesting that observing CT-optimal touch might increase CSE. However, mu oscillatory responses during action observation are thought to reflect the mirroring of tactile components rather than the motor features (e.g. velocity) of action execution (Coll et al. 2015). Conversely, prior research on pain perception indicated a decrease in motor cortical output during both self- and vicarious experiences of pain (Farina et al. 2003; Avenanti et al. 2005), although there are contrasting findings (Galang and Obhi 2021). Interestingly, larger CSE inhibition and greater mu rhythm suppression are both held as proxies of sensorimotor resonance to vicarious pain (Cheng et al. 2008; Vitale et al. 2023). Based on these findings on vicarious pain perception, we anticipated a decrease in MEP amplitude during the observation of CT-optimal (slow) touch compared to both static and fast touch.

A secondary objective of our study was to explore the relationship between individual differences in touch experiences and attitudes, as well as in interoceptive awareness (the awareness of the connection between body sensations and emotional states), and motor resonance to interpersonal touch. Prior research has shown that personal affective experiences of CT stimulation, altered due to factors such as CT-fiber deafferentation or environmental deprivation, can impact the perception of vicarious touch (Morrison et al. 2011b; Devine et al. 2020). Additionally, interoceptive processing, which involves the integration and elaboration of internal body signals, may play a significant role in shaping the perception of external stimuli (Seth and Friston 2016) and particularly the perception of vicarious touch (Gillmeister et al. 2022). Because of the unique neural pathway that links the pIC to CT afferents in the skin, affective touch might indeed be considered an interoceptive modality. Variations in interoception have been reported to influence how individuals provide touch (Bytomski et al. 2020) and to modulate vicarious responses to touch in somatosensory areas (Adler and Gillmeister 2019; Rigato et al. 2019b; Bellard et al. 2023). Therefore, here, we aimed to verify whether interoceptive awareness may influence motor resonance to vicarious touch.

## Materials and methods

### Participants

Based on the effect size (*n^2^_p_* = 0.16) reported by a previous TMS-MEP paper on interpersonal-action observation and adopting a similar design (Betti et al. 2022), considering a repeated-measures (RM) ANOVA model with two muscles, two body sites, and three stroking velocities, an a priori power analysis using the G^*^Power 3.0.10 software with the “as in SPPS” option (Faul et al. 2007) indicated a target sample size of 28, with 80% power and alpha set at 0.05. Thirty participants completed the experiment in our laboratory at Liverpool John Moores University (LJMU). The data from two participants were discarded since they reported, at a follow-up debriefing session, a lack of naivety about the research stimuli and question, which could alter the results. Therefore, the final sample consisted of 28 participants (15 females, 13 males; age mean = 27.5 years, SD = 5.7). Recruitment was through posters, social media advertisements, and email invitations to our research panel lists. Inclusion criteria were: (i) having normal or corrected to normal vision (with glasses/contact lenses), (ii) being right-handed, (iii) no history of neurological or psychiatric disorders, and (iv) no chronic pain or skin diseases. Participants were screened for TMS exclusion criteria through a safety screening questionnaire (Rossi et al. 2009; Rossi et al. 2021), with no contraindications reported. Compensation included a £10 gift voucher and Sona-systems points for undergraduate psychology students. All procedures were approved by the LJMU Research Ethics Committee (reference number: 22/PSY/078) and were in keeping with the Declaration of Helsinki, with written informed consent obtained.

### General procedure

Eligible volunteers completed online a preliminary TMS screening questionnaire (Rossi et al. 2021). On the scheduled date, participants recompleted the safety questionnaire to exclude any arising contraindications to TMS. Seated in a recliner with their right arm on a pillow, participants watched video clips on a 28″ monitor (resolution 1,920 × 1,200 pixels, refresh rate 60 Hz) positioned at approximately 100 cm. MEPs were recorded during and before/after the experimental task with video presentation. At the end of the experimental session, participants also completed self-report questionnaires on demographics, interoceptive awareness (Multidimensional Assessment of Interoceptive Awareness [MAIA]), and touch attitudes/experiences (Touch Experiences and Attitudes Questionnaire [TEAQ]). Right-handedness was verified using the Edinburgh Handedness Inventory (Oldfield 1971). The procedure took about 70 min, followed by debriefing.

### Stimuli and task structure

During the experimental task, participants were presented with interpersonal touch video clips taken from previous studies from our lab (Trotter et al. 2018a; Bellard et al. 2022, 2023). A recent behavioral study using the same video clips documented that the appreciation of both toucher- and touchee-referred vicarious touch is specifically attuned to CT-optimal touch (Butti et al. 2024). The 6-s-long videos displayed both male and female actors applying touch with their right hand to other female and male actors (i.e. 4 actor sex combinations). Touch was delivered with three different velocities: 5 cm/s as CT-optimal stroking and 0 cm/s (static) and 30 cm/s (fast) as non-CT-optimal velocities. While static touch may not elicit motor resonance as effectively as dynamic touch, this condition provided a necessary control condition to compare against slow and fast stroking conditions, thus enabling a clearer understanding of the motor system’s response to the absence of motion (Gallese and Sinigaglia 2011). Moreover, static touch represents a natural and ecologically valid modality of tactile stimulation during interpersonal interactions (Ali et al. 2023). Touch was applied on the hand dorsum and on the palm, which represent body sites with different densities of CT fibers, respectively, a hairy and a glabrous skin site. Importantly, these two body regions were selected as they were matched in terms of size and observed movements. Considering 4 actor-sex combinations, 3 velocities and 2 body sites, a total of 24 videos represented all possible conditions.

Before the video presentation, participants were instructed that they would be presented with interpersonal touch videos while receiving spTMS. Crucially, instructions required participants to pay attention to the biological sex of the toucher because in some trials they would be asked to verbally indicate whether the toucher was male or female. This explicit sex judgment task was thought to engage participants during video presentation and to prompt them to focus on the actor delivering interpersonal touch and, thus, on the touch action. A verbal, rather than a motor response was chosen to avoid MEP contamination due to hand-response preparation (Betti et al. 2022).

**Fig. 1:**
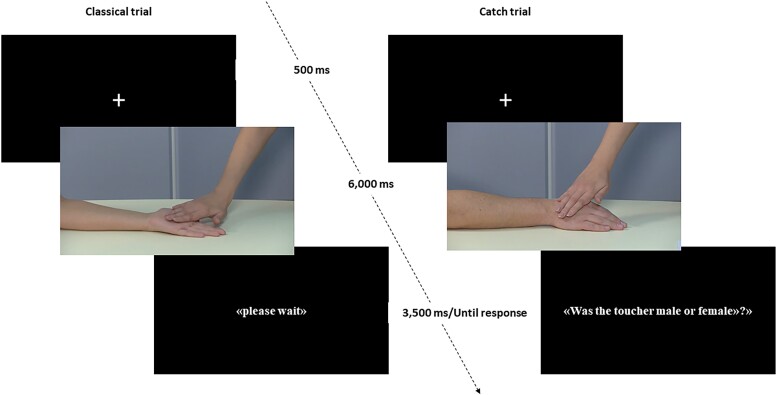
Trial structure. Examples and timeline of classical and catch trials.

Each trial started with a fixation cross lasting 500 ms, followed by the 6-s-long interpersonal touch videos. The spTMS was administered in the last second of video presentation, to ensure participants were exposed to full action unfolding (details below). At the end of the video, “classical” trials displayed a black background screen reporting “please wait” in white letters for 3500 ms. This way, the whole trial lasted 10 s. Conversely, in “catch” trials the last slide reported the question “Was the toucher male or female?”, written in white letters on a black background. Participants had to answer verbally (i.e. “male,” “female”) and the experimenter recorded verbal responses by pressing the “m” or “f” tab on a wireless keyboard. Participants were asked to provide their answers only at the end of the videos to prevent contamination of MEPs by verbal responses, which could induce changes in the CSE of hand muscles (Meister et al. 2003; Onmyoji et al. 2015). MEPs were recorded during both classical and catch trials. The response accuracy in catch trials (mean = 95%, SD = 5%) confirmed that participants were engaged in the task. The response slide lasted until a response was recorded and its duration across participants was roughly equivalent to the “please wait” slide in classical trials (mean = 2919 ms, SD = 467 ms). Examples of a classical and a catch trial are reported in Fig. 1.

On the basis of a 2 muscle × 3 velocity × 2 body site within-subject design, and to obtain 32 observations per cell, a total of 192 trials were presented. Of them, 48 were catch trials (25%). This way, each of the 24 video stimuli was displayed six times in classical trials and two times in catch trials. The order of video and trial presentation was completely randomized. The E-Prime 3 software (Psychology Software Tools, Pittsburgh, PA, USA) controlled task administration and response recording.

### TMS and MEP recording procedure

TMS was performed by means of a 70-mm figure-eight stimulation coil (Magstim D70 Alpha Flat Coil), connected to a Magstim SuperRapid2 Stimulator (The Magstim Company, Carmarthenshire, Wales, UK) producing a magnetic field up to 0.8T at the surface of the coil. MEPs were recorded simultaneously from the extensor carpi radialis (ECR) and the first dorsal interosseous (FDI) of the right limb. These two muscles represent, respectively, a proximal and a distal muscle involved in interpersonal touch movements (Schieppati et al. 1996; van Kuijk et al. 2009). The decision to measure the ECR muscle activity was rooted in the need to explore whether motor resonance to observed touch would manifest differently across muscles that vary in their proximity to the site of observed action and their involvement in typical touch-related movements (Naish et al. 2014). The ECR muscle, being a proximal muscle, is involved in the extension of the wrist and thus plays a role in the overall motor pattern associated with reaching and touching actions. Measuring the activity of the ECR muscle, in conjunction with a distal muscle like the FDI, allowed the study to assess whether motor resonance is generalized across the limb or if it is more pronounced in muscles more directly involved in fine motor control related to touch. A greater modulation in the distal muscle (i.e. FDI) would suggest the importance of motor feedback from hand muscles in understanding the toucher’s intentions (Betti et al. 2022). Conversely, similar MEPs from both muscles would indicate a more generalized motor reactivity, likely associated with affective processing (Lepage et al. 2010). Surface Ag/AgCl disposable electrodes (1 cm diameter) were placed in a belly–tendon montage for each muscle. Electrode positions were determined by palpation during maximum voluntary contraction for each muscle, with reference electrodes placed over the ipsilateral interphalangeal joint for the FDI and over the ulnar styloid process for the ECR, while the ground electrode was positioned over the right elbow.

Prior to MEPs recording, participants were tested for their resting motor threshold (rMT), which is the minimum stimulus intensity able to evoke MEPs from both the muscles with amplitude of at least 50 μV in 50% of 10 trials (rMT mean = 72%, SD = 14%). Given that the ECR typically has a higher rMT compared to the FDI muscle (Wu et al. 2002), the ECR muscle was prioritized when setting the individual rMT. Moreover, for each participant, the optimal position of the coil was determined by moving the coil in approximately 0.5 cm steps around the scalp position corresponding to the left motor hand area and by delivering TMS pulses at constant intensity until recording maximal amplitude MEPs from both muscles. The determined position was marked on a tight-fitting cap wore by participants, and the coil was held securely to the scalp ensuring the magnetic pulses were only given to the area of interest. In line with previous studies (Borgomaneri et al. 2015; Amoruso and Urgesi 2016), the coil was placed tangentially on the scalp, with the handle pointing backward and approximately 45° lateral from the midline.

During task presentation, spTMS was administered to left M1 according to one of five time-delays after video onset (5,100, 5,200, 5,300, 5,400, 5,500 ms). These delays were chosen to ensure that participants were exposed to the full unfolding of kinematics. While this methodological choice limited the detection of potential different stages of CSE modulation, it allowed for a more accurate comparison by accounting for variations in the onset of touch actions across the videos. Delay variability prevented any anticipatory effect of the stimulation (Tran et al. 2021; Betti et al. 2022). The stimulation intensity was set at 120% of the individual rMT. Before and after the experimental task, MEPs were recorded during 10 baseline trials in which participants were presented with a fixation cross.

A Biopac MP-36 system (BIOPAC Systems, Inc., Goleta, CA) was used for signal amplification, band-pass filtering (5 to 1,000 Hz) and digitalization (sampling rate 10,000 Hz). The TMS pulse was also recorded as a digital input channel starting when the TMS was triggered and lasting 15 ms. TMS stimulation and EMG recording were controlled by the E-Prime 3 software. Offline analysis of EMG data was executed by means of the AcqKnowledge software (BIOPAC Systems, Inc., Goleta, CA).

### MAIA

The MAIA Mehling et al. 2012) is a 32-item questionnaire that provides eight dimensions of interoceptive bodily awareness: noticing (4 items), not distracting (3 items), not worrying (3 items), attention regulation (7 items), emotional awareness (5 items), self-regulation (4 items), body listening (3 items), and trusting (3 items). Answers are recorded on a 6-point Likert scale ranging from 0 = “Never” to 5 = “Always,” with some questions being reversed scored. Each subscale is scored by the average of the corresponding items. The MAIA questionnaire was found to have a good internal consistency (Cronbach α = 0.90) (Valenzuela-Moguillansky and Reyes-Reyes 2015).

### TEAQ

The 37-item version of the TEAQ (Trotter et al. 2018a; Trotter et al. 2018b) is a self-report that assesses current and childhood experiences of positive touch and an individual’s attitude toward interpersonal touch. Questions are answered using a 5-point Likert scale ranging from 1 = “Disagree strongly” to 5 = “Agree strongly,” with negatively worded questions being reverse scored. Five subscales are calculated by the average of the corresponding items: attitude to friend and family touch (7 items), attitude to intimate touch (10 items), childhood touch (8 items), attitude to self-care (7 items), and current intimate touch (5 items). Good internal consistency was reported for the TEAQ (Cronbach α = 0.93).

### Data handling and statistical analysis

For each muscle and condition, MEPs were calculated as peak-to-peak EMG signal (in mV) from the end of the digital input representing the TMS pulse for the following 40 ms. With the aim to control for muscle preactivation and artifact, trials in which the peak-to-peak signal from 70 to 10 ms before the TMS pulse was higher than mean + 2 SD were discarded and excluded from further analyses. Across all subjects and for both muscles excluded trials were less than 10% (ECR: mean = 3.7%, SD = 1.9%; FDI: mean = 4.6%, SD = 1.7%), thus ensuring EMG data reliability. Changes in basal CSE during the experiment were examined by comparing the pre- and postbaseline raw MEPs through paired-sample *t*-tests (two-tailed). Postbaseline MEPs of two subjects were not recorded due to technical issues. Regarding the experimental task, for each participant and separately for the two muscles, the raw MEP amplitude of each trial was normalized according to the distribution of all trials (Z-score). Transformation into Z-scores was thus calculated using the individual MEP mean and SD for each muscle across the experimental trials. This transformation was chosen to control for interindividual variability and to insert the two muscles in the same analysis. The Z-scores were inserted into an RM-ANOVA with 2 muscles (ECR, FDI), 2 body sites (hand dorsum, palm), and 3 velocities (static, CT-optimal, fast) as within-subject variables. Post hoc analysis was performed using Duncan’s test correction, which reduces the size of the critical difference depending on the number of steps separating the ordered means and is optimal for testing in the same design effects that may have different sizes (McHugh 2011).

Since we anticipated CSE modulation based on stroking velocities, specifically a decrease in MEPs for CT-optimal touch, a delta index was calculated to measure the mean difference in Z-scores between CT-optimal and non-CT-optimal velocities. The formula was: *[(CT-optimal—fast) + (CT-optimal—static)]/2*. Although this formula was partially aligned with prior behavioral research from our laboratory (Bellard et al. 2022; Butti et al. 2024), since no subjective ratings were collected in this study, we named the index Affective Touch Sensitivity (ATS) to avoid any misunderstanding. Higher negative values would indicate greater MEP suppression (i.e. decrease in Z-scores) for CT-optimal compared to non-CT-optimal velocities, while higher positive values would indicate greater MEP facilitation (i.e. increase in Z-scores) for CT-optimal touch. The ATS was calculated separately for each muscle as we expected differences between proximal and distal muscles (van Kuijk et al. 2009). A Pearson’s correlation analysis, adopting Bonferroni correction for multiple comparisons, was then conducted between the ATS indexes and interoceptive awareness and individual differences in touch attitudes and experiences. Given that previous behavioral studies have found significant associations between interoceptive trusting, attitudes toward touch from friends and family, and vicarious touch perception (Bellard et al. 2022; Butti et al. 2024), these subscales of the MAIA and TEAQ were included in the correlation analysis. For the TEAQ, the childhood touch experience subscale was added to investigate whether early touch experiences might influence motor resonance to vicarious touch, similar to how they impact subjective ratings of vicarious pleasantness (Devine et al. 2020). Given the interplay between interoceptive awareness, emotional processing, and vicarious experiences (Seth and Friston 2016), the MAIA emotional awareness scale was also incorporated into the correlation analysis.

The effect size was estimated as partial eta squared (*η^2^_p_*) for ANOVA (small: 0.01, medium: 0.06, large: 0.14) and as Cohen’s *d* for pairwise comparisons (small: 0.2, medium: 0.5, large: 0.8). A significance threshold of *P* = 0.05 was set for all statistical analyses. Data are reported as mean ± SEM. The analyses were performed using the STATISTICA software (StatSoft Inc., version 8). The ggplot2 package version 3.4.3 (Wickham 2016) of the R software (version 4.3.1; R Foundation for Statistical Computing) was used to perform data visualization.

## Results

### MEP modulation

The comparisons between the MEP amplitudes recorded as pre- and post-task baseline yielded no significant results either for the ECR (*t_25_* = 0.56, *P* = 0.582) or the FDI (*t_25_* = 1.10, *P* = 0.283), probing that the TMS per se did not change basal MEPs across the experiment. The normalized MEPs for each condition are reported in Table 1.

**Table 1:** MEP *Z*-score means (±SEM) for the ECR and FDI muscles according to the body site on which touch was delivered and stroking velocities.

**Muscle**	**Body site**	**Velocity**	**Z-score**
ECR	Hand dorsum	Static	0.035 ± 0.027
ECR	Hand dorsum	CT-optimal	−0.065 ± 0.034
ECR	Hand dorsum	Fast	0.011 ± 0.027
ECR	Palm	Static	0.047 ± 0.039
ECR	Palm	CT-optimal	−0.032 ± 0.031
ECR	Palm	Fast	0.009 ± 0.031
FDI	Hand dorsum	Static	0.031 ± 0.036
FDI	Hand dorsum	CT-optimal	−0.058 ± 0.028
FDI	Hand dorsum	Fast	−0.023 ± 0.036
FDI	Palm	Static	0.030 ± 0.033
FDI	Palm	CT-optimal	−0.043 ± 0.032
FDI	Palm	Fast	0.054 ± 0.035

**Fig. 2:**
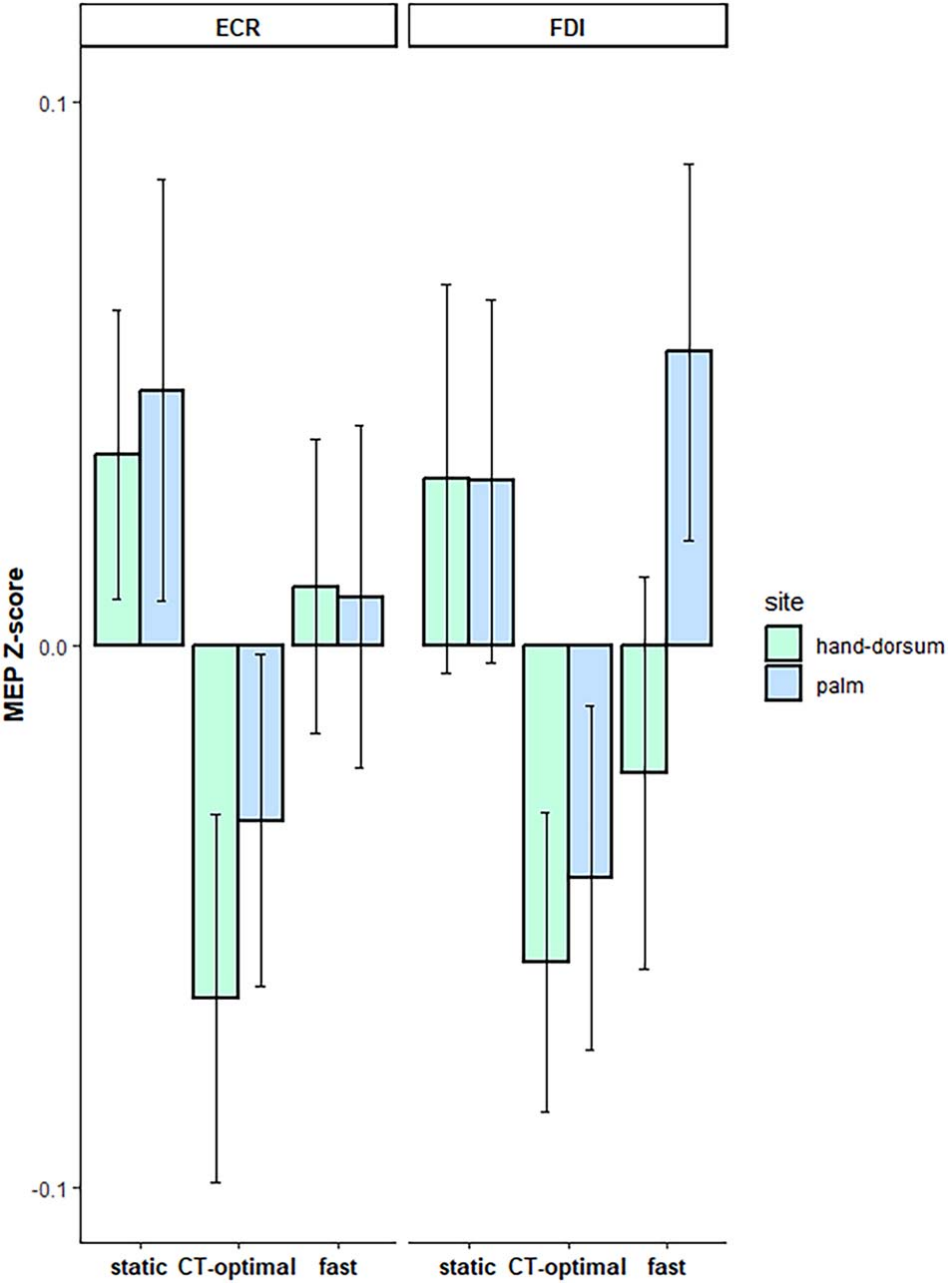
Barplot of MEP amplitudes. The two panels represent the *Z*-standardized MEPs recorded from the ECR and FDI muscles. Error bars represent SEM.

The RM-ANOVA revealed a significant main effect of velocity, with a large effect size (*F_2,54_* = 5.09, *P* = 0.009, *n^2^_p_* = 0.16). Post hoc analysis indicated significant, medium-to-large differences in MEP amplitudes between static and CT-optimal (slow) touch (0.036 ± 0.016 vs.−0.050 ± 0.013; *P* = 0.005, Cohen’s *d* = 1.10), and between CT-optimal (slow) and fast stroking (0.013 ± 0.018; *P* = 0.029, Cohen’s *d* = 0.75). Such a difference was not significant between static and fast touch (*P* = 0.406, Cohen’s *d* = 0.25). Neither the main effects of muscle and body site nor the interaction effects were significant (all *F* < 3.50, all *P* > 0.071) (see Supplementary Table 1 for further details on the results). To sum up, these findings pointed to lower MEP amplitudes for CT-optimal (slow) velocity compared to non-CT-optimal velocities, detected in both the muscles and regardless of the body site (Fig. 2).

### Correlation analyses

**Fig. 3:**
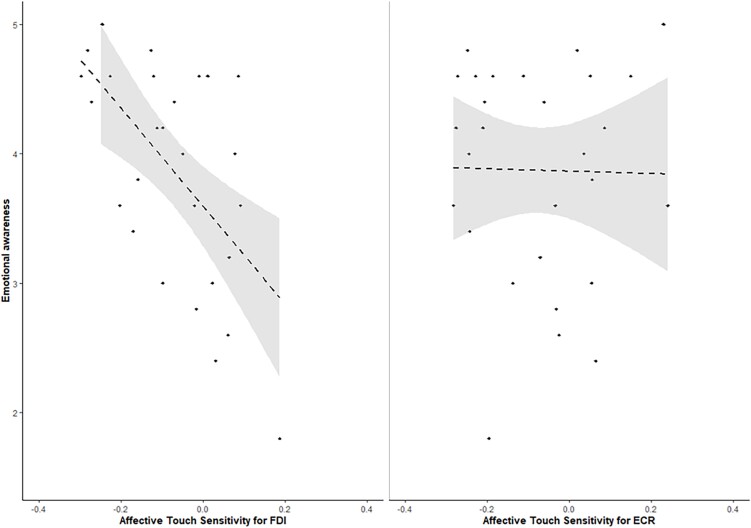
Scatterplots of correlation between emotional awareness (MAIA subscale) and ATS index for the ECR and FDI muscles. Dots represent observations; the dotted black line represents the regression line; the shaded gray area represents 95% confidence interval.

A strong, negative correlation emerged between the ATS index for the FDI muscle and the MAIA emotional awareness scale (*r* = −0.58, *P* = 0.001). The same correlation was not significant for the ECR muscle (*r* = −0.02, *P* = 0.930). These results indicated that the more participants used their bodily cues to be aware of their emotional states, the greater the MEP suppression in the FDI for CT-optimal compared to non-CT optimal velocities (Fig. 3).

The ATS indexes did not significantly correlate with the MAIA trusting scale (all *r* < |0.35|, all *P* > 0.073), nor with the selected TEAQ scales (all *r* < |0.22|, all *P* > 0.282) (see Supplementary Table 2 for further details on the correlation results across all questionnaire scales).

## Discussion

This study investigated whether observation of interpersonal touch applied at CT-optimal vs. non-CT-optimal velocities might affect motor resonance, defined as the covert activation of the motor system, measured via MEPs. We also tested the direction of motor resonance to observed touch, specifically whether it results in CSE facilitation, leading to larger MEPs compared to baseline, or in CSE suppression, manifesting as smaller MEPs. For this purpose, we used spTMS to measure CSE while participants observed video clips depicting interpersonal touch events with different stroking velocities. We then explored whether individual differences in touch experiences and attitudes, as well as in interoceptive awareness, are related to motor resonance to interpersonal touch.

Motor resonance to interpersonal touch was modulated by stroking velocities, with a decrease in CSE when vicarious touch was delivered at CT-optimal (slow) velocity compared to both static and fast touch. These results suggest that motor cortex activation can distinguish CT-optimal stroking during the observation of reception and delivery of interpersonal touch (Morrison et al. 2011a), resulting in a modulation of CSE in the arm muscles. The sensitivity of the primary motor cortex to the observation of affective touch may seem in contrast with prior fMRI research reporting that passively observing interpersonal touch increased the activation in somatosensory and socio-cognitive networks but not in the M1 (Lee Masson et al. 2018). However, that study did not systematically explore a preference for CT-optimal touch, which we have observed here.

Motor resonance during action observation typically manifests as facilitation of CSE, which is held to reflect the internal simulation of the model’s movement (Naish et al. 2014). Thus, regarding the modulation of motor resonance to interpersonal touch, the question is why the CSE decreases specifically for CT-optimal stroking. Interestingly, the decrease in MEP amplitude during the observation of CT-optimal (slow) compared to both static and fast touch represents an opposite pattern compared to the typical inverted-U-shaped trend widely documented for CT-fiber firing and pleasantness rating (Löken et al. 2009; McGlone et al. 2014; Ackerley 2022). A potential explanation for this result is that motor resonance suppression may facilitate somatosensory simulation of the observed (affective) touch, a mechanism that would help individuals capture the hedonic value of CT-optimal stroking and “resonate” with others’ affective experiences of being touched (Schaefer et al. 2012; Peled-Avron et al. 2016; Lee et al. 2019). This hypothesis is in line with extensive research documenting the contribution of somatosensory networks to vicarious touch (Keysers et al. 2004; Keysers et al. 2010; Bolognini et al. 2011; Bolognini et al. 2012; Peled-Avron et al. 2019; Rigato et al. 2019a). Notably, a similar inhibition of CSE has been documented while participants witnessed the delivery of a painful tactile stimulation (i.e. a syringe injection) to the hand of a model compared to when they observed the delivery of a neutral tactile stimulus (i.e. Q-tip touch) (Avenanti et al. 2005; Avenanti et al. 2006; Avenanti et al. 2010; Vitale et al. 2023). Conversely, the same experimental conditions were shown to be associated with an activation of S1 and posterior parietal cortex (Bufalari et al. 2007; Costantini et al. 2008), but no activation differences were found in the primary motor cortex (Lamm et al. 2011; Fallon et al. 2020). This literature on pain observation suggests that the simultaneous inhibition of primary motor representations and activation of somatosensory areas may play a role in encoding the sensory and affective features of the observed bodily sensations (Bufalari and Ionta 2013).

In a similar vein, the somatosensory simulation might be prioritized over the motor simulation of the observed touch as it is the touchee’s experience to determine whether the stroking is perceived as pleasant, adequate to the context, and matched to the toucher’s purpose, thus providing more information than vicarious execution to infer affective and social values conveyed by interpersonal touch (Kirsch et al. 2018; Sailer and Leknes 2022). This prioritization of somatosensory simulation might hint at inhibitory connectivity between S1 and M1 (Solodkin et al. 2004), which has been shown to contribute to motor inhibition during motor imagery (Guillot et al. 2012). Accordingly, a recent study (Oldrati et al. 2021) showed that inhibiting neural activity of S1 with repetitive TMS boosted CSE facilitation during motor imagery. The decrease in CSE observed here might thus reflect the processing of somatosensory features in S1 (Valchev et al. 2017), leading to the inhibition of motor simulation in M1. However, while these processes may seem complementary and closely related, the present study did not provide direct evidence of causal inhibitory interactions between M1 and somatosensory areas during the observation of affective touch. Future research is needed to investigate these interactions further.

No differences were found between hairy (i.e. hand dorsum) and glabrous (i.e. palm) skin sites, which are differently innervated by CT fibers (Ackerley et al. 2014b), but see also (Watkins et al. 2021). On one hand, this result confirms our experimental setup that participants were exposed to similar movements on both skin sites, which were chosen to be matched in terms of size, thus ruling out that our findings could be influenced by different kinematics for touch delivery on the hand dorsum or the palm. On the other hand, this finding supports the idea that the motor system is attuned to the execution of CT-optimal touch but not to the body site on which the touch was delivered. Accordingly, we can speculate that this information may be more effectively retrieved by somatosensory simulation of the observed touch, as evidenced by previous research showing dissociable somatosensory responses to touch on hairy and glabrous skin (Schirmer et al. 2022). Moreover, overlapping somatosensory activation patterns between felt and observed touch were found to encode information about the location of touch (Smit et al. 2023).

Higher reliance on bodily cues to be emotionally aware was associated with greater motor suppression for CT-optimal compared to non-CT-optimal velocities. This correlation could be interpreted within the earlier mentioned speculation, namely, that suppression of motor resonance to affective touch might aid in understanding the touchee’s feelings during vicarious interpersonal touch. Since simulation processes are rooted in their own embodied representations of the observed action (Gallese and Ebisch 2013), it could be that participants more aware of their bodily signals are better able to perceive the observed touch as if they were the touchee and may benefit from a suppression of motor resonance to facilitate somatosensory simulations. Consistently, previous research has shown that individuals with higher levels of interoceptive awareness exhibit greater responses in somatosensory areas for vicarious touch perception (Adler and Gillmeister 2019). In a related study, Bellard et al. (2023) reported that, after inhibition of the somatosensory area, the higher the liking to be touched, the higher the levels of emotional awareness. Interoceptive awareness may thus partially account for the variability in vicarious touch experience and the inhibitory motor output found here. Overall, our result adds to previous literature pointing to a link between vicarious touch and interoceptive awareness (Schaefer et al. 2013; Lamm et al. 2015; Peled-Avron et al. 2016; Peled-Avron et al. 2019; Smit et al. 2023).

It should be noted though that this correlation was found to be significant for the FDI but not for the ECR muscle. The FDI, as a distal muscle and part of the hand, is directly involved in touch actions. Therefore, it might provide more feedback about the motor intentions of the toucher, which could be decreased for CT-optimal touch to facilitate somatosensory simulation. On the other hand, the main analysis did not highlight an interaction effect of muscle with velocity, suggesting that the quadratic relation between MEP amplitudes and velocities was similar for the two muscles. Previous research has indicated that a nonmuscle-specific modulation of motor resonance might reflect rapid and automatic processing related to social and emotional functioning (Lepage et al. 2010). The absence of this muscle specificity, however, could be due to the methodological choice to assess CSE well after the video onset. While this choice ensured that participants were exposed to the full unfolding of the action, it limited our ability to disentangle different stages of CSE modulation (Naish et al. 2014).

To the best of our knowledge, this was the first study investigating motor resonance to observations of affective touch. Nevertheless, limitations must be acknowledged when interpreting the results of this study. First, the speculations advanced to explain our results need to be confirmed and further explored in wider samples. Specifically, the hypothesis that a decrease in CSE would facilitate understanding the somatosensory consequences of the observed touch should be directly tested in future research, e.g. testing somatosensory-evoked potentials vs. MEPs (Galvez-Pol et al. 2020). In a similar vein, this speculation does not exclude that the motor suppression mechanism described here might influence vicarious experience of touch at higher representational levels rather than at early sensorimotor cortices (Kilteni et al. 2021; Smit et al. 2023). Although we manipulated stroking velocities as the main parameter to distinguish CT-optimal vs. non-CT-optimal touch, we cannot exclude that other touch features, such as the perceived naturalness of the touch (Lee Masson and Op de Beeck 2018), might also influence motor resonance. Including control comparison conditions, such as observing CT-optimal speed movements without tactile interaction or observing CT-optimal touch delivered on inanimate objects, would help disentangle which motor and tactile features of CT-optimal touch are mapped by the motor cortex. The absence of these control conditions limits the extent to which we can claim a selective sensitivity of the motor cortex to affective touch. To disentangle specific stages of CSE modulation and muscle-specific effects, future studies may explore the distinct contributions of emotional reactivity and motor simulation to vicarious touch by investigating different time windows (Borgomaneri et al. 2012; Finisguerra et al. 2021). Moreover, even though the videos adopted here were previously validated and adopted to assess vicarious pleasantness, we did not ask participants to rate pleasantness for the observed touch. How motor simulation processes may influence mechanisms of touch execution appraisal should be examined in future research. Although the adoption of videos showing static touch represented an ecological condition and aligned with previous research (Bellard et al. 2022; Bellard et al. 2023; Butti et al. 2024), in the future, comparing slow, CT-optimal touch with videos displaying very slow touch (e.g. 0.5 cm/s) could provide further insights into the CT-specific modulation of motor resonance. An a priori power analysis was conducted to determine the appropriate sample size for the primary aim of the study, which was to compare motor resonance to touch delivered at CT-optimal and non-CT-optimal velocities. A medium-to-high association was observed between interoceptive awareness and MEP modulation; however, the study may have been underpowered to detect smaller correlations. Lastly, previous research consistently documented sex differences in the perception of affective touch (Russo et al. 2020). Although relatively balanced, the sample of this study was too small to explore sex and gender differences in motor resonance to vicarious touch, which should be explored in future research.

In conclusion, this study provides novel insights into the neural mechanisms underlying motor resonance in response to the observation of affective touch. Our findings highlight a unique modulation of motor system activity, particularly a decrease in CSE when observing CT-optimal touch as opposed to non-CT-optimal velocities. This suggests a selective sensitivity of the motor cortex to affective, CT-optimal touch, potentially facilitating somatosensory simulation over motor simulation of the observed touch. The significant correlation found between emotional awareness and motor resonance further underscores the intricate relationship between interoceptive awareness and the processing of vicarious touch experiences. These results contribute to a deeper understanding of the complex interplay between motor and somatosensory systems in social touch perception and emphasize the importance of affective touch in human social interactions. Future research should aim to explore these mechanisms further, particularly how these processes integrate with the emotional and cognitive aspects of touch perception. This study not only advances our understanding of the neural basis of social touch but also opens new avenues for investigating the role of affective touch in social cognition and emotional empathy.

## Supplementary Material

Supplementary_material_bhae441

## Data Availability

All the relevant data are freely available from OSF at the following weblink: https://osf.io/sravy/?view_only=c4cb8d3827cf4891a95678b61f074ad9.
