# Supplementary material for: Neurophysiological evidence of motor contribution to vicarious affective touch
Source: Cereb Cortex. 2024 Nov 6;34(11):bhae441. doi: 10.1093/cercor/bhae441 (PMC11540462; doi:10.1093/cercor/bhae441)
Supplement: Supplementary_material_bhae441 [file supplementary_material_bhae441.pdf]

Supplementary table 1. Results of the RM-ANOVA on MEP Z-scores. The significant effect is highlighted in bold.

**Effect**	***F***	***p***	$\boldsymbol{n^2_p}$
Muscle	3.50	0.072	0.11
Body site	0.93	0.344	0.03
Velocity	**5.09**	**0.009**	**0.16**
*Muscle*Body site*	0.16	0.689	0.01
*Muscle*Velocity*	0.09	0.911	0.00
*Body site*Velocity*	0.14	0.871	0.01
*Muscle*Body site*Velocity*	0.92	0.404	0.03

Supplementary table 2. Pearson's correlations between the Affective Touch Sensitivity (ATS) indexes and all subscales of the TEAQ and MAIA questionnaires. The selected subscales and significant correlation are highlighted in bold.

**Questionnaire scales**	**Affective Touch Sensitivity (ATS)**			
	ECR		*FDI*	
	r	*p*	*r*	*p*
**MAIA**				
Noticing	0.34	0.073	-0.31	0.104
Not distancing	0.16	0.409	0.29	0.141
Not worrying	0.12	0.548	0.18	0.362
Attention regulation	0.28	0.146	-0.10	0.614
Emotional awareness	-0.02	0.930	**-0.58**	**0.001**
Self-regulation	0.14	0.476	-0.37	0.052
Body listening	0.18	0.368	-0.32	0.093
Trusting	-0.12	0.541	-0.34	0.074
**TEAQ**				
Friend and family touch	-0.09	0.658	-0.04	0.827
Intimate touch	-0.05	0.796	-0.05	0.818
Childhood touch	0.10	0.612	0.21	0.283
Self-care	-0.26	0.179	0.30	0.120
Current intimate touch	-0.13	0.511	-0.06	0.771
